# Foliar application of licorice-wolfberry derived nanomaterials enhances soybean heat tolerance through maintaining reactive oxygen species homeostasis

**DOI:** 10.3389/fpls.2026.1795808

**Published:** 2026-06-12

**Authors:** Jie Liu, Bingqin Qi, Lingfeng Bao, Tingyong Mao, Lili Yang, Hengbin Zhang, Desheng Wang, Jiahao Liu, Yong Zhan, Yunlong Zhai

**Affiliations:** 1 College of Agriculture, Tarim University, Alar, China; 2 Key Laboratory of Genetic Improvement and Efficient Production for Specialty Crops in Arid Southern Xinjiang of Xinjiang Corps, Tarim University, Alar, China; 3 Crops Research Institute, Xinjiang Academy of Agricultural and Reclamation Sciences, Shihezi, China

**Keywords:** heat stress, licorice - wolfberry nanomaterial, nitrogen metabolism, reactive oxygen species, soybean

## Abstract

High-temperature stress severely limits soybean growth and yield, particularly in regions with extreme climates such as southern Xinjiang. This study aimed to investigate the regulatory mechanisms by which foliar application of licorice- and goji berry-derived mesoporous self-assembled nanomaterials (LW-CNs) modulates the heat tolerance of soybean seedlings. Soybean seedlings were treated via foliar spraying, and their physiological and biochemical responses were analyzed 3 days after heat stress treatment. The results indicated that LW-CNs treatment significantly improved the plant morphology of soybeans, increasing fresh weight, dry weight, leaf area, and SPAD values, while simultaneously enhancing net photosynthetic rate, transpiration rate, and stomatal conductance. Regarding the antioxidant system, foliar application of LW-CNs significantly increased antioxidant enzyme activities (superoxide dismutase, SOD: 619.5 ± 12.6 U g^−1^ vs. 531.2 ± 2.0 U g^−1^; peroxidase (POD): 110.2 ± 0.5 U g^−1^ vs. 71.8 ± 3.7 U g^−1^). Furthermore, LW-CNs activated the ascorbic acid-glutathione (AsA-GSH) cycle, significantly increasing APX and GR activities as well as GSH and AsA contents, thereby effectively maintaining reactive oxygen species (ROS) homeostasis. Regarding nitrogen metabolism, LW-CNs increased NH_4_^+^ content and the activities of key enzymes such as GS, GOGAT, GDH, GPT, and GOT, thereby promoting nitrogen uptake and assimilation. In summary, our findings indicate that foliar application of LW-CNs during the seedling stage is an effective strategy for enhancing soybean heat tolerance and contributes to a deeper understanding of how nanobiotechnology regulates ROS.

## 1 Introduction

Climate change poses a significant threat to plant survival and agriculture. As a major abiotic stressor, rising temperatures can severely disrupt plant growth and yield (Lippmann et al., 2019). This phenomenon is particularly evident in soybeans; for every 1 °C increase in global temperature, soybean yields are projected to decline by 3.1%, highlighting heat stress as a major limiting factor in global soybean production (Zhao et al., 2017; Liu et al., 2019). In southern Xinjiang, located in the interior of the Eurasian continent, seasonal temperatures have shown a steady upward trend over the past few decades (Xu et al., 2010; Peng et al., 2014), with temperatures exceeding 40 °C recorded across various regions (China Meteorological Data Network: https://data.cma.cn). Studies have shown that under high-temperature conditions, the closure of plant leaf stomata leads to increased intercellular carbon dioxide concentrations, thereby reducing photosynthetic efficiency (Cao et al., 2019; Loka et al., 2020; Li et al., 2024).

High-temperature stress leads to excessive accumulation of reactive oxygen species (ROS), such as hydrogen peroxide (H_2_O_2_) and superoxide anion (O_2_^·−^), in plants, thereby triggering oxidative damage and causing elevated levels of malondialdehyde (MDA), which in turn impairs the structure and function of cell membranes (Nishizawa et al., 2006; Mishkind et al., 2009). To counteract this process, plants operate an antioxidant defense system to scavenge ROS. This system includes peroxidases (POD), superoxide dismutases (SOD), catalases (CAT), ascorbate peroxidases (APX), and associated reductases, which act synergistically to scavenge ROS and mitigate oxidative damage (Arora and Bhatka, 2017; Cao et al., 2018). Studies have shown that as temperatures rise, leaf area and dry weight of aboveground biomass decrease, while maintaining a steady state of reactive oxygen species is beneficial for plant growth (Jumrani and Bhatia, 2017; López-Vargas et al., 2020). High temperatures can affect the antioxidant system of soybeans, leading to a reduction in pod number and pod weight (Du et al., 2022).

Nitrogen (N) is an essential nutrient for plants. Studies have shown that under high-temperature stress conditions, nitrogen uptake in soybeans and rice is inhibited (Das et al., 2017; Wang et al., 2025a). Because of the reduction in the activity of enzymes involved in nitrogen metabolism, and alterations in the GS-GOGAT cycle; that are key factors contributing to the decline in crop nitrogen metabolism (Hungria and Kaschuk, 2014; Fortunato et al., 2023). Thus, the activity of antioxidant enzymes, as well as nitrogen uptake, transport, and assimilation, are crucial for enhancing soybean heat tolerance.

Nanobiotechnology is an emerging technology that can maintain the equilibrium of reactive oxygen species (ROS) under non-biological stress (Zhao et al., 2023). Currently, studies on enhancing the non-biological stress tolerance of crops by maintaining ROS homeostasis through nanobiotechnology include the use of cerium oxide (CeO_2_), zinc oxide (ZnO), iron oxide (FeO) and Se nanomaterials (An et al., 2020; Liu et al., 2022; Mazhar et al., 2022; Gu et al., 2024; Jiang et al., 2025). It is worth noting that the exogenous application of nano-selenium materials under drought stress can enhance the activity of plant antioxidant enzymes, regulate reactive oxygen species levels, and maintain cellular integrity (Huang et al., 2026). Nanomaterials can mitigate the damage plants sustain under various stress conditions through a variety of mechanisms. Hossain et al (Hossain et al., 2015), suggested that many nanoparticles are released into the environment, which in turn can have an impact on inhibiting plant growth. Therefore, selecting a biocompatible and safe nanomaterial is essential for boosting soybean yield.

In recent years licorice and wolfberry derived complex nanomaterials (LW-CNs) possesses low toxicity, chemical stability, and antibacterial properties, and it has the ability to maintain the balance of reactive oxygen species (ROS) within plants under non-biological stress conditions (Qiu et al., 2024; Wang et al., 2025b). The research on LW-CNs mainly focuses on the application of increasing the germination rate of wheat under salt stress and the underlying mechanism behind it. However, the biological effects of LW-CNs on soybeans under high temperature stress are still not well defined.

This study aims to clarify the alleviating effect of LW-CNs on high-temperature stress in soybean seedlings, emphasizing its effective role in ROS homeostasis by regulating the antioxidant enzyme defense system, as well as the response of nitrogen metabolism-related enzymes, to high-temperature stress. The outcomes of this study will provide a theoretical basis for the development of sustainable agriculture.

## 2 Materials and methods

### 2.1 Synthesis of LW-CNs

Following the method described in our previous report (Qiu et al., 2024). Specifically, 200 mg of licorice powder or goji berry powder was weighed and placed in a high-pressure reactor containing 10 mL of deionized water, then heated at 160 °C for 6 hours. After the reaction, the resulting mixture was centrifuged at 12,000 rpm for 1 hour, and the supernatant was collected, yielding licorice-based mesoporous spheres (Li-MSs) and goji berry-based carbon dots (Wo-CDs), respectively. To further prepare LW-NCs, 100 mg each of licorice powder and goji berry powder were dispersed in 10 mL of deionized water, transferred to a high-pressure reactor, and heated at 160 °C for 6 hours. After the reaction was complete, centrifugation was performed under the same conditions (12,000 rpm, 1 hour), and the supernatant containing LW-NCs was collected. All prepared nanomaterials were stored at 4 °C for use in subsequent experiments. LW-CNs were applied at a concentration of 200 mg L^−1^, with a DLS-measured particle size of 42.2 ± 8.2 nm and a zeta potential of −19.6 ± 1.5 eV (Wang et al., 2025b). All samples in this paper represent independent biological replicates.

### 2.2 Plant materials and growth conditions

Soybean seeds of the cultivar ‘Xin dadou. 26’ were sown in white plastic pots (diameter 39.5 cm, height 10 cm). Thirty-five seeds were sown per pot and germinated in a growth chamber under controlled conditions: 25–30 °C, 55–60% relative humidity, and a 14/10 h (light/dark) photoperiod. Upon seedling emergence, uniformly germinated seedlings were selected and transferred to Hoagland nutrient solution for hydroponic cultivation until full cotyledon expansion. Following foliar application of LW-CNs, plants were maintained in darkness for 3 h and then subjected to high-temperature stress. Heat stress was imposed by exposing the plants to 45/40 °C (day/night) under a 14/10 h photo period for 3 days in a growth chamber. Two treatments were applied: (1) control plants (foliar application of distilled water), and (2) LW-CNs plants. After the stress treatment, leaves were collected, flash-frozen in liquid nitrogen, and stored at −80 °C for subsequent physiological analyses.

### 2.3 Measurement of leaf photosynthetic parameter

Parameters including net photosynthetic rate (A), stomatal conductance (Gs), intercellular CO_2_ concentration (Ci) and transpiration rate (E) were measured using a portable gas exchange analyzer (Li-6800 Licor, Lincoln, NE, USA) at the 3^rd^ of day treatment. Measurements were performed in a leaf chamber with a CO_2_ concentration of 400 ppm, a mean temperature of 25 °C, a flow rate of 500 μmol sec^-1^ and light level of 1500 umol m^-2^ sec^-1^.

### 2.4 ROS-scavenging ability assay of LW-CNs

At the 3^rd^ of day of heat stress, O_2_^-^ scavenge rate in soybean leaves was determined following a previously described method (Wang et al., 2025b) and calculated using a SOD assay kit (WST^-1^) (Nanjing Jiancheng Bioengineer, Nanjing, China). First, O_2_^-^ were produced by xanthine and xanthine oxidase reactions. The generated O_2_^-^ reacted with WST^-1^ to make a water-soluble formazan dye, which was detected by measuring the absorbance at 450 nm. LW-CNs were added (final nanoparticle concentration of 200 mg L^-1^) to the mixture of xanthine and xanthine oxidase and then incubated at 37 °C for 30 min. The 300 μL suspension was measured at 450 nm using a microplate spectrophotometer (Epoch, Biotek, USA).

### 2.5 Determination of protein content and soluble sugar content

After three days of heat stress, the protein content of soybean leaves treated with LW-CNs and control was measured separately. The protein content was determined using a protein content assay kit (Mengxi Biomedical Technology Co., Ltd., Jiangsu, China), following the manufacturer’s instructions.

To determine the soluble sugar content fresh soybean leaves (0.2 g) were flash-frozen with liquid nitrogen and ground into a fine powder. The powder was extracted with 5 mL of 80% (v/v) ethanol solution at 80 °C for 30 min in a water bath, with intermittent vortex mixing (3 times during extraction). After centrifugation at 12,000 ×g for 10 min, the supernatant was collected. A 0.5 mL aliquot of the supernatant was vigorously vortexed with 3 mL of freshly prepared anthrone-sulfuric acid reagent (0.2 g anthrone dissolved in 100 mL ice-cold concentrated sulfuric acid). The mixture was heated precisely for 10 min in a boiling water bath, then immediately cooled to room temperature in an ice bath. Absorbance was measured at 620 nm against a reagent blank, and soluble sugar content was calculated based on a glucose standard curve.

### 2.6 Determination of reactive oxygen species content, antioxidant enzyme activity, and malondialdehyde content

The LW-CNs treated soybean leaves and control were sampled to determine the ROS content, antioxidant enzyme activity, and MDA content. The H_2_O_2_ and O_2_^·−^ contents in leaves were measured using ROS detection kits. Briefly, the H_2_O_2_ content of soybean leaves was measured using an H_2_O_2_ assay kit (Mengxi Biomedical Technology Co., Ltd., Jiangsu, China). To determine the O_2_^·−^ content, an O_2_^·−^ assay kit was used (Mengxi Biomedical Technology Co., Ltd., Jiangsu, China). Peroxidase (POD), catalase (CAT), superoxide dismutase (SOD), and malondialdehyde (MDA) detection kits (Mengxi Biomedical Technology Co., Ltd., Jiangsu, China) were used to determine the POD, CAT, and SOD activity and MDA content, respectively.

### 2.7 Determination of key enzymes and metabolites of the ascorbate–glutathione cycle

The LW-CNs treated and control soybeans leaves under heat stress were used to determine key enzymes and metabolites in AsA-GSH cycle. Ascorbate peroxidase (APX), glutathione reductase (GR), monodehydroascorbate reductase (MDHAR), and dehydroascorbate reductase (DHAR) activities were determined using APX, GR, MDHAR, and DHAR assay kits (Mengxi Biomedical Technology Co., Ltd., Jiangsu, China), respectively, following the manufacturer’s instructions.

Dehydroascorbic acid (DHA) can be converted into reduced ascorbic acid, which can react with ferric ions (Fe^3+^) to form ferrous ions (Fe^2+^), reacting with red phenanthroline to form a red chelate with an absorption peak at 534 nm. The DHA content in the sample was determined by measuring the reduced ascorbic acid before and after the sample reduction. To determine the oxidized glutathione (GSSG) content, 2-VP was combined with GSH to exclude GSH interference. GR catalyzed the redox reaction between GSSG and NADPH so that GSSG reduce to GSH. GSH then reacted with 2-nitrobenzoic acid (DTNB) to form a chromogenic substance, which reacts to the GSSG in the sample. To determine the GSH content, DTNB was reacted with GSH to form a complex, showing a characteristic absorption peak at 412 nm. The reduced ascorbic acid content in soybean leaves was determined using the AsA assay kit (Mengxi Biomedical Technology Co., Ltd., Jiangsu, China), following the manufacturer’s instructions.

### 2.8 Determination of key enzymes and metabolites of the nitrogen metabolism

At the 3^rd^ of day of heat stress, the LW-CNs treated soybean leaves and control were used to determine key enzymes and metabolites in nitrogen metabolism.

Leaf NH_4_^+^ content was determined spectrophotometrically using a modified protocol (Bräutigam et al., 2007). Briefly, 100 mg of freeze-dried leaf powder was extracted in 1 mL of 100 mM HCl, followed by the addition of 500 μL of chloroform. The mixture was vortexed for 15 min at 4 °C and centrifuged at 12,000 × g for 10 min at 8 °C. The aqueous phase was transferred to a new tube containing 50 mg of activated charcoal, mixed thoroughly, and centrifuged again at 20,000 × g for 5 min at 8 °C. The resulting supernatant was diluted 1:1 (v/v) with 100 mM HCl. For colorimetric analysis, 20 μL of the diluted extract was mixed with 100 μL of a reagent containing 1% (w/v) phenol and 0.005% (w/v) sodium nitroprusside, followed by the addition of 100 μL of a solution containing 1% (v/v) sodium hypochlorite and 0.5% (w/v) sodium hydroxide. The mixture was incubated at 37 °C for 30 min, and absorbance was measured at 620 nm.

Glutamine Synthetase (GS) activity was assayed following the method of (Yu and Zhang, 2012). Glutamine:α-Ketoglutarate Aminotransferase (GOGAT) activity was measured using the protocol described by (Singh and Srivastava, 1986). Glutamate Dehydrogenase (GDH) activity was determined by monitoring the reduction of NAD^+^ (deaminating activity, NAD-GDH) or the oxidation of NADH (aminating activity, NADH-GDH) according to (Groat and Vance, 1981). Glutamic-Pyruvic Transaminase (GPT) and Glutamic-Oxalacetic Transaminase (GOT) activities were analyzed using commercial assay kits (Mengxi Biomedical Technology Co., Ltd., Jiangsu, China) following the manufacturer’s instructions.

## 3 Results

### 3.1 Influence of LW-CNs on soybean phenotype

**Figure 1:**
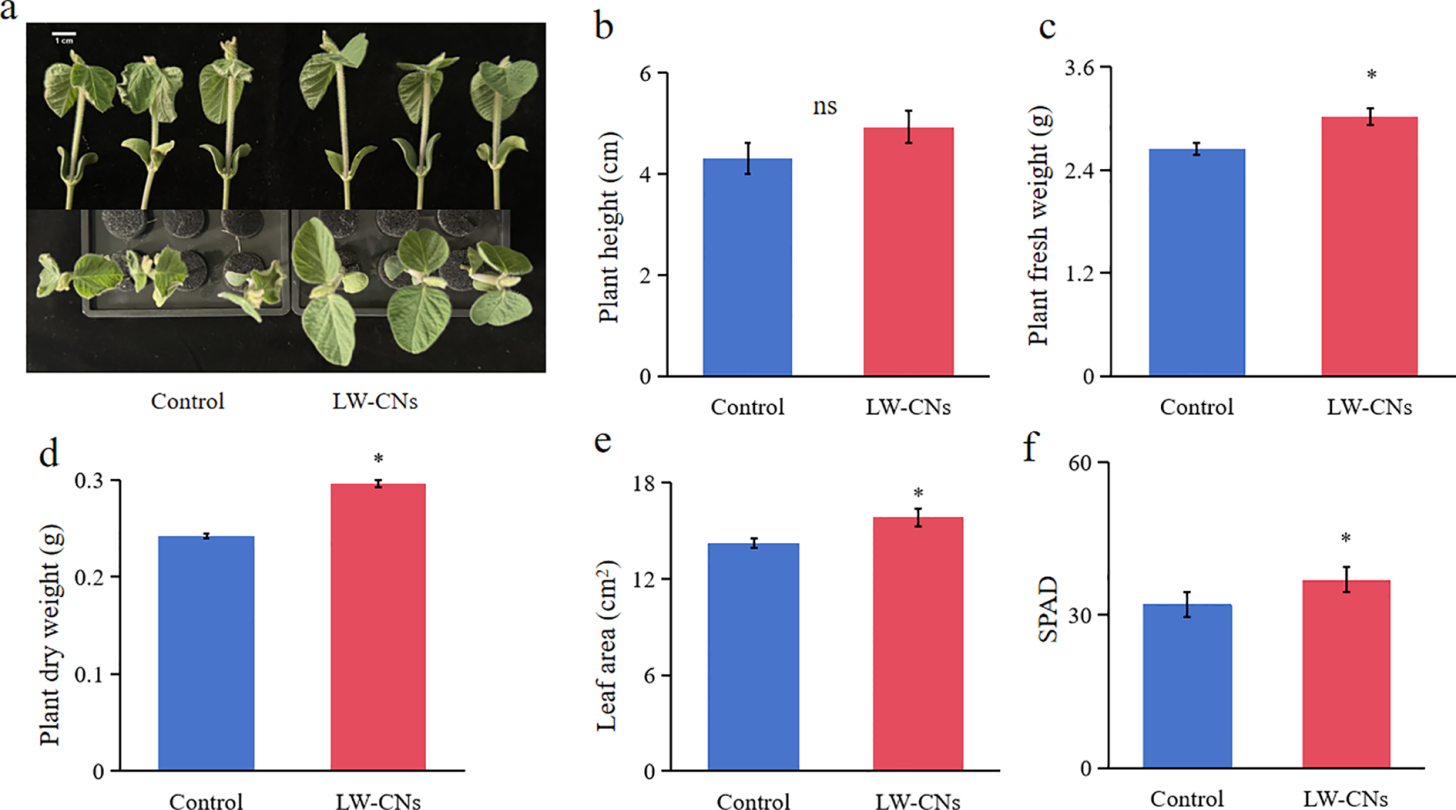
Phenotype of 3 days soybean seedling under heat stress with and without LW-CNs treatment. **(A)** Growth phenotype for seedling; **(B)** plant height, Mean ± SE (n = 6); **(C)** plant fresh weight, Mean ± SE (n = 6); **(D)** plant dry weight, Mean ± SE (n = 6); **(E)** leaf area, Mean ± SE (n = 6); **(F)** SPAD, Mean ± SE (n = 6); A comparison between treatments was performed by independent sample t tests (two tailed) in **(B–F)**. * indicate significance at p ≤ 0.05 levels.

To determine the effects of LW-CNs on soybean’s heat tolerance, soybean phenotype was compared between LW-CNs-treated and control under 40°C heat stress. As shown in **Figures 1A, LW**-CNs treatment resulted in a better growth phenotype than the control group. Compared to the control group, the plant dry weight and fresh weight in the LW-CNs-treated group increased significantly by 25.0% and 31.9%, respectively (**Figures 1C, D**). The leaf area and SPAD in the LW-CNs treatment also increased by 16.5% and 17.5%, respectively, compared with the control group (**Figures 1E, F**).

### 3.2 Effects of LW-CNs on soybean measurement of leaf photosynthetic parameters

**Figure 2:**
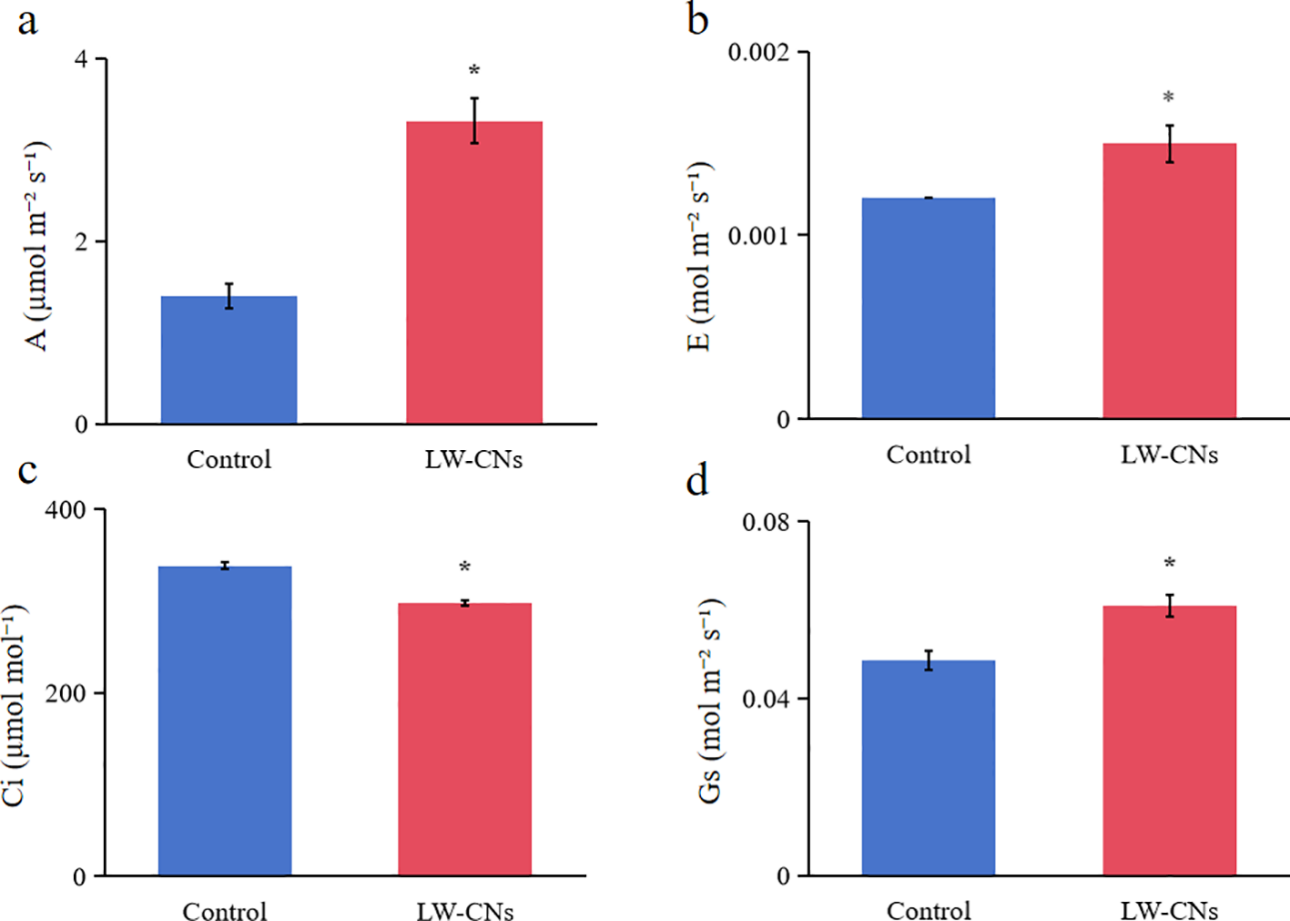
Measurement of leaf photosynthetic parameters 3 days soybean seedling under heat stress with and without LW-CNs treatment. **(A)** photosynthetic rate; **(B)** transpiration rate; **(C)** intercellular CO_2_ concentration; **(D)** stomatal conductance; A comparison between treatments was performed by independent sample t tests (two tailed) in **(A–D)**. * indicate significance at p ≤ 0.05 levels. Mean ± SE (n = 3).

As shown in **Figure 2**, compared to the control group, the A, E, and Gs in treated leaves significantly increased by 57.9, 20.0, and 20.2%, respectively as compared to control. The Ci concentration activities significantly reduced by 12.1%, under LW-CNs treatment. These findings suggest that LW-CNs treatment enhanced the leaf photosynthetic activity.

### 3.3 Influence of LW-CNs on soybean protein and soluble sugar content

**Figure 3:**
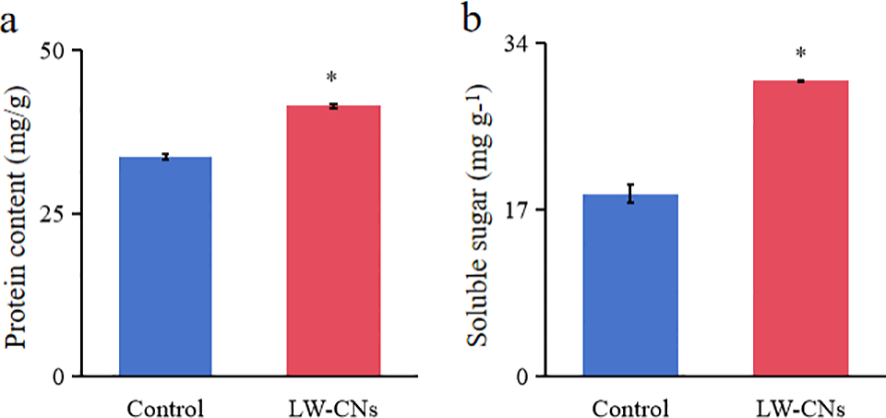
Protein and soluble sugar content 3 days soybean seedling under heat stress with and without LW-CNs treatment. **(A)** protein content; **(B)** soluble sugar content; A comparison between treatments was performed by independent sample t tests (two tailed) in **(A, B)**. * indicate significance at p ≤ 0.05 levels. Mean ± SE (n = 3).

As shown in **Figure 3**, LW-CNs treatment resulted in a higher protein and soluble sugar content than the control group. The protein content in the LW-CNs-treated group increased significantly by 23.3% (**Figure 3A**) and the soluble sugar content increased significantly by 52.8% (**Figure 3B**).

### 3.4 Effects of LW-CNs on antioxidant enzyme activity and ROS homeostasis

**Figure 4:**
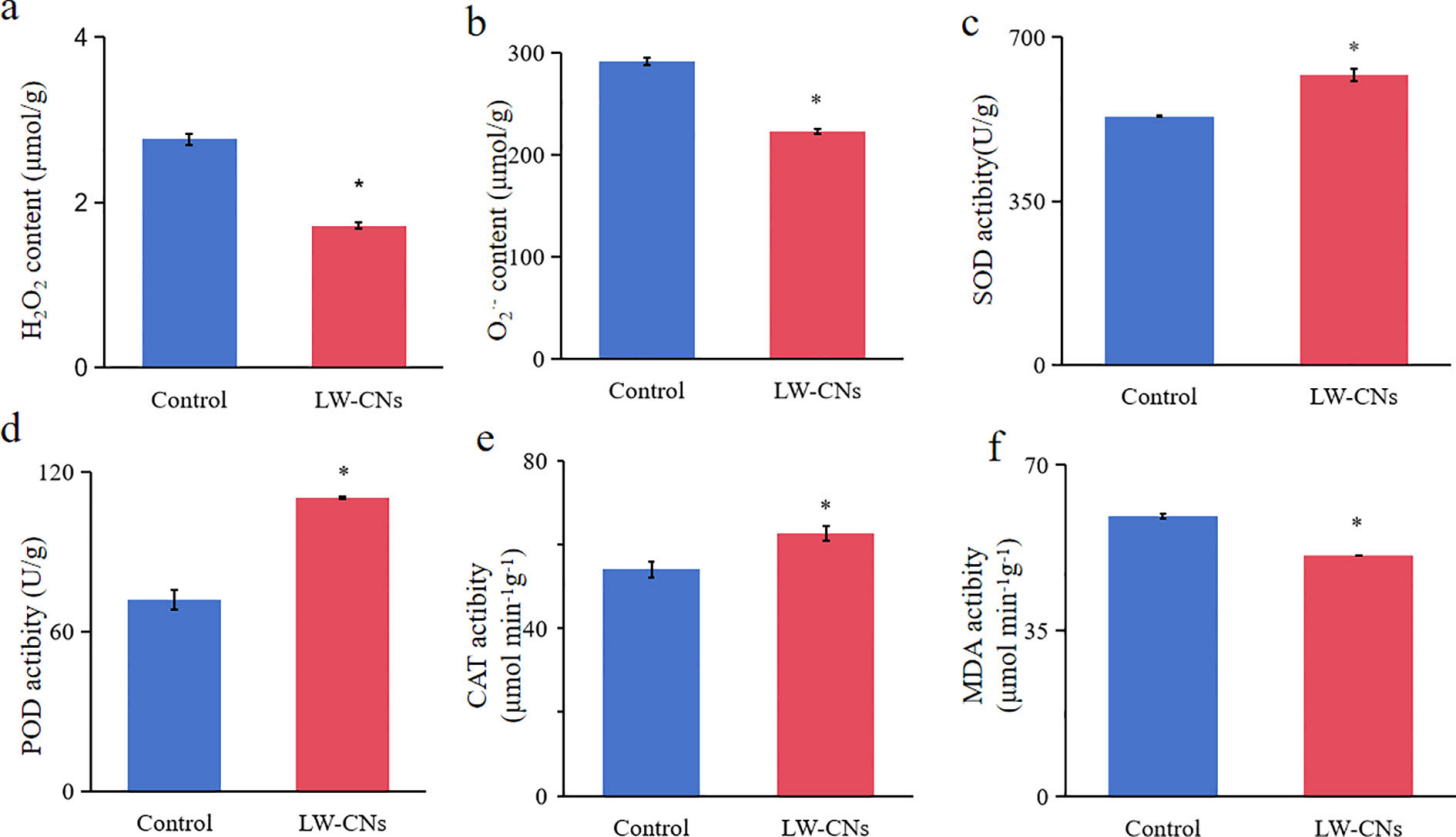
ROS content and antioxidase enzyme activity of 3 days soybean seedling under heat stress with and without LW-CNs treatment. **(A)** H_2_O_2_ content; **(B)** O_2_^·−^ content; **(C)** Superoxide dismutase (SOD) activity; **(D)** peroxidase (POD) activity; **(E)** catalase (CAT) activity; **(F)** malondialdehyde (MDA) content. A comparison between treatments was performed by independent sample t tests (two tailed) in **(A–F)**. * indicate significance at p ≤ 0.05 levels. Mean ± SE (n = 3).

In this study compared to the control group, the O_2_^·−^, H_2_O_2_, and MDA contents in soybean leaves treated with LW-CNs significantly decreased by 31.1, 38.0, and 16.2%, respectively (**Figure 4**). As shown in **Figure 4**, the SOD, POD, and CAT activities significantly increased by 14.2, 34.8, and 13.8%, respectively, under LW-CNs treatment. LW-CNs treatment enhanced the enzyme activity. These findings suggest that LW-CNs alleviates excess ROS accumulation in soybean plants by activating antioxidant enzymes.

### 3.5 Effect of LW-CNs on the AsA-GSH cycle in soybean under heat stress

**Figure 5:**
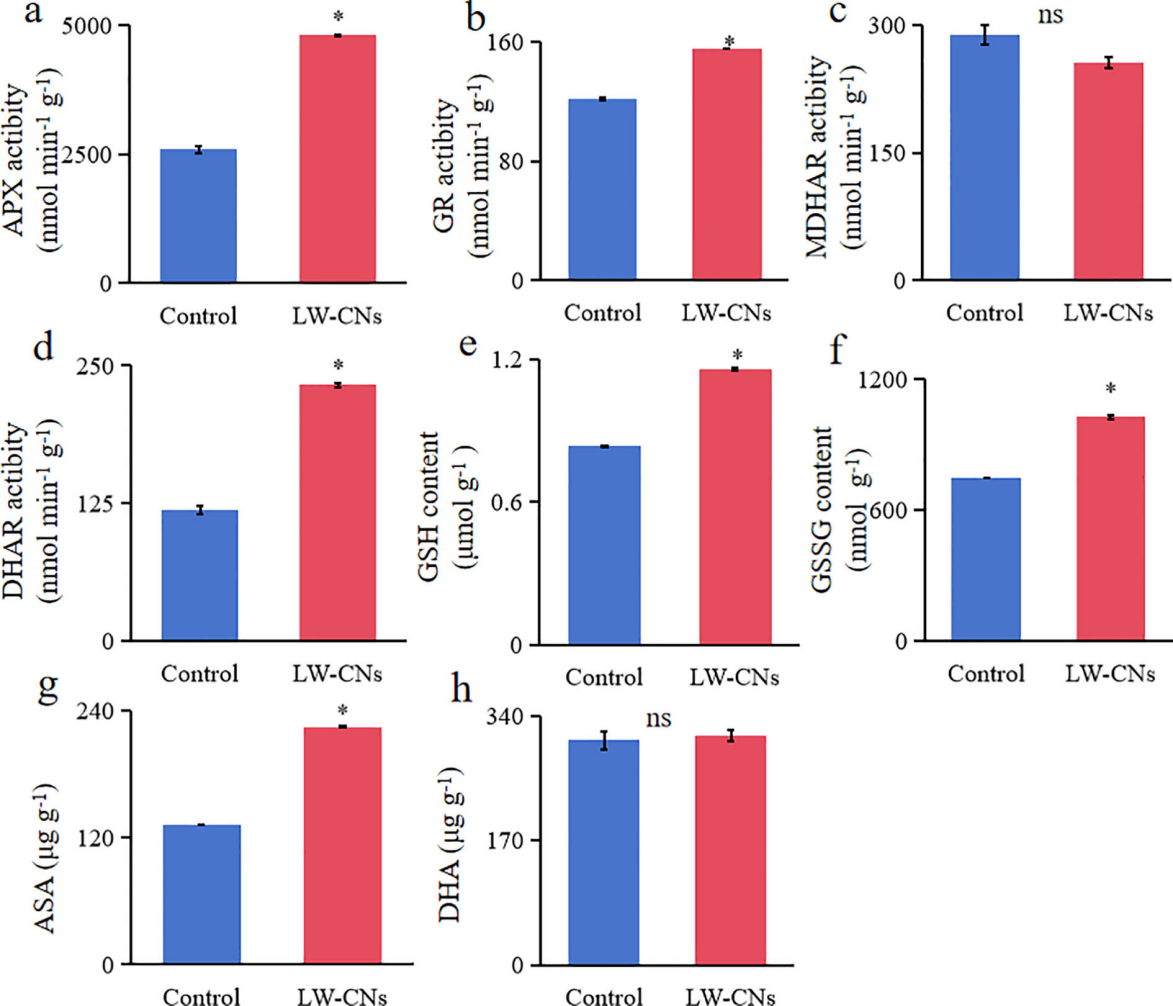
Key enzymes activity and metabolites content in the AsA-GSH cycle of 3 days soybean seedling under heat stress with and without LW-CNs treatment. **(A)** Ascorbate peroxidase (APX) activity; **(B)** glutathione reductase (GR) activity; **(C)** monodehydroascorbate reductase (MDHAR) activity; **(D)** dehydroascorbate reductase (DHAR) activity; **(E)** glutathione (GSH) content; **(F)** glutathione, oxidized (GSSG) content; **(G)** ascorbic acid (AsA) content; **(H)** Dehydroascorbate (DHA) content. A comparison between treatments was performed by independent sample t tests in **(A–H)**. * indicate significance at p ≤ 0.05 levels. Mean ± SE (n = 3).

The activities of APX and GR significantly increased by 85.1 and 28.1%, respectively (**Figure 5A, B**). In contrast, the MDHAR activities showed no significant differences (**Figure 5C**). GSH and GSSG, as critical antioxidants in the AsA-GSH cycle, exhibited significantly increased by 38.5 and 37.5%, respectively in the LW-CNs treatment compared with control group (**Figures 5E, F**). Additionally, under LW-CNs treatment, the content of ASA increased by 69.8% compared to the control (**Figure 5G**). While the DHA content showed no significance for LW-CNs treated and control group (**Figure 5H**).

### 3.6 Effect of LW-CNs on the nitrogen metabolism in soybean under heat stress

**Figure 6:**
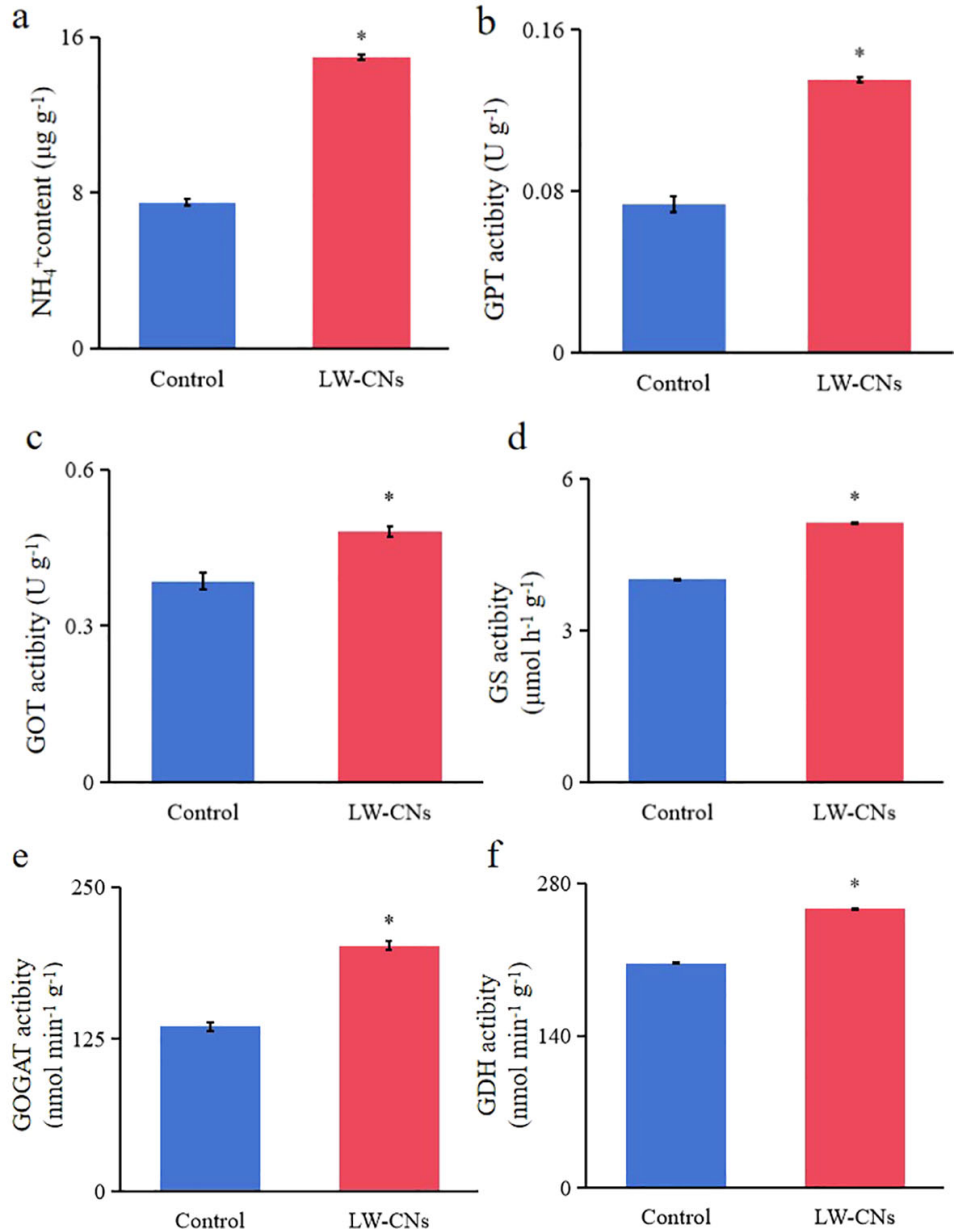
Key enzymes activity and metabolites content in the nitrogen metabolism of 3 days soybean seedling under heat stress with and without LW-CNs treatment. **(A)** NH_4_^+^ content; **(B)** Glutamic-pyruvic transaminase (GPT) activity; **(C)** Glutamic-oxalacetic transaminase (GOT) activity; **(D)** Glutamine synthetase (GS) activity; **(E)** Glutamine:α-ketoglutarate aminotransferase (GOGAT) activity; **(F)** Glutamate dehydrogenase (GDH) activity; comparison between treatments was performed by independent sample t tests in **(A–F)**. * indicate significance at p ≤ 0.05 levels. Mean ± SE (n = 3), ns indicates no significant difference.

In terms of nitrogen metabolism, the NH_4_^+^ content in LW-CNs-treated leaves was notably higher than that in the control group (**Figure 6A**). This increase was accompanied by elevated activities of key enzymes involved in nitrogen assimilation and amino acid metabolism. Specifically, the activities of GPT (**Figure 6B**), GOT (**Figure 6C**), GS (**Figure 6D**), GOGAT (**Figure 6E**), and GDH (**Figure 6F**) were all significantly higher in the LW-CNs treated group. These findings suggest that LW-CNs treatment promoted nitrogen uptake and assimilation in soybean leaves, potentially by enhancing the activities of enzymes involved in ammonia incorporation and amino acid synthesis. The significant increases in enzyme activities indicate that LW-CNs may have stimulated the metabolic pathways related to nitrogen utilization, leading to improved nitrogen use efficiency in soybean plants.

## 4 Discussion

### 4.1 LW-CNs help soybeans maintain their plant morphology and enhance photosynthesis under high-temperature conditions

Stunted plant growth due to high-temperature stress is caused by a reduction in photosynthetic capacity (Hu et al., 2023). Research findings indicated that engineered nanoparticles can elevate the photosynthetic efficiency of crops by binding to Rubisco enzymes (Routier et al., 2023). Simultaneously, numerous studies have been conducted on the utilization of nanomaterials to augment the photosynthetic rate of crops under adverse stress conditions (Liu et al., 2024; Zhang et al., 2024). For example, cerium dioxide (CeO_2_) nanoparticles have demonstrated the ability to effectively enhance the photosynthesis of rapeseed under salt stress, thereby facilitating their growth (Rossi et al., 2017). Cheng et al, successfully enhanced the light energy utilization efficiency of crops through the application of carbon dots (CDs), leading to an increase in the photo-assimilation rate (Cheng et al., 2023). These findings suggest that leveraging nanomaterials to enhance crop photosynthesis is both feasible and has broad applicability.

The results showed that after foliar spraying of LW-CNs on soybean, actively regulated important physiological processes, thus soybean seedlings and leaves could maintain a better plant morphology under high-temperature stress. It is worth noting that LW-CNs enhanced photosynthetic performance, protects the integrity of photosynthetic pigments, and thereby increases biomass. However, as far as we know, research on using nanomaterials to promote photosynthesis in soybeans under high-temperature conditions is still relatively limited. This might be attributed to the fact that high-temperature pressure can directly damage the structure of photo-cooperative enzymes in soybeans. In addition, further research is needed to fully understand how nanomaterials protect the photosynthetic system of soybeans under high-temperature stress conditions.

### 4.2 When soybeans are subjected to high-temperature stress, LW-CNs maintains reactive oxygen species homeostasis in plants by enhancing antioxidant capacity through the AsA-GSH cycle

Excessive accumulation of reactive oxygen species under high-temperature stress exerts toxic effects on plants and leads to the inactivation of enzymes within plant cells (Suzuki and Mittler, 2006). Liu et al (Liu et al., 2021), used CeO_2_ to maintain the ROS homeostasis in cotton under salt stress and enhance its salt tolerance, which was due to the increased activities of superoxide dismutase (SOD), peroxidase (POD), and catalase (CAT). In contrast, Khan et al (Khan et al., 2022), found that the CAT activity in rapeseed seeds primed with CeO_2_ decreased, despite an improvement in the salt tolerance of rapeseeds. In this study, the application of LW-CNs increased the activity of POD and CAT, thereby enhancing soybean tolerance to high-temperature stress. This effect may be attributed to the antioxidant properties of LW-CNs. Moreover, enzymes such as SOD, APX and GSH also participate in the antioxidant defense system (Yildiztugay et al., 2016; Imran et al., 2021).

**Figure 7:**
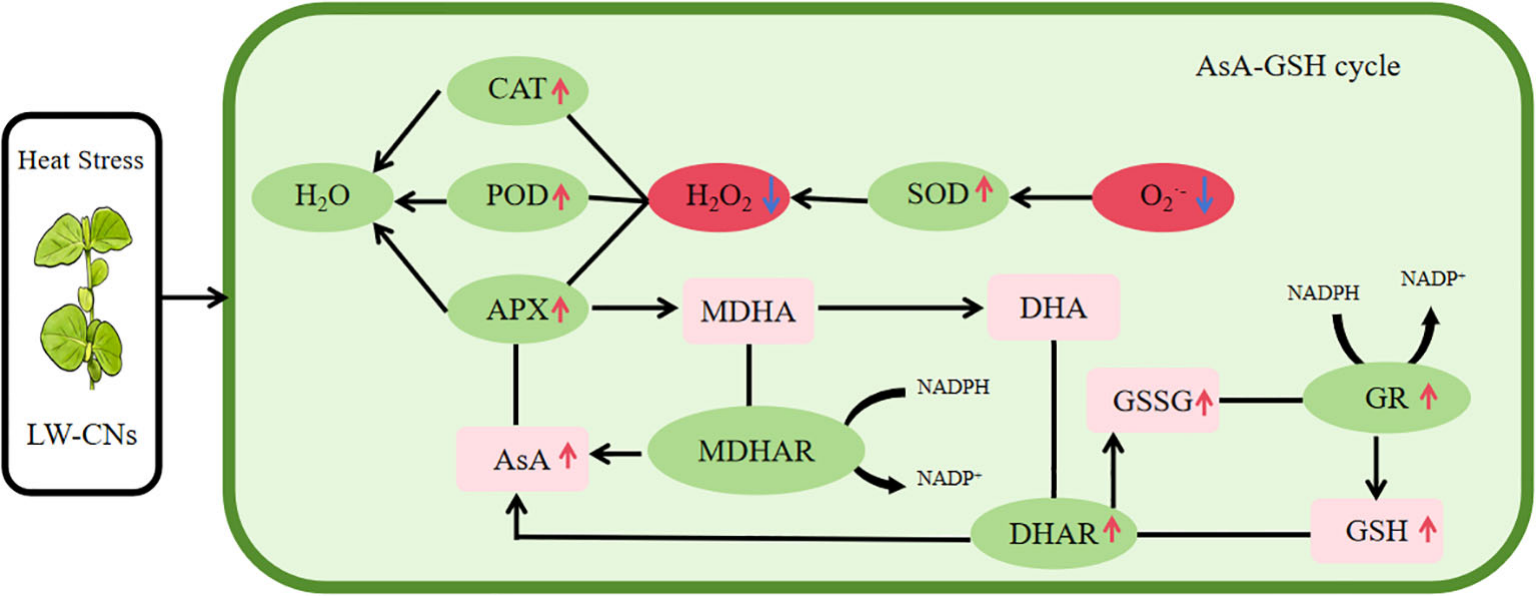
The schematic diagram illustrates the mechanism by which LW-CNs foliage spray treatment effectively maintains reactive oxygen species (ROS) homeostasis in soybean plants through two synergistic pathways: enhancing the functional capacity of antioxidant enzymes and preserving the integrity of the ascorbate-glutathione (AsA-GSH) metabolic cycle.

APX is the core enzyme of the AsA-GSH cycle and plays a crucial role in H_2_O_2_ metabolism (Ishikawa and Shigeoka, 2008). Research by Shan et al (Shan et al., 2011), indicated that modulating the activity of ascorbate peroxidase (APX), glutathione reductase (GR), and dehydroascorbate reductase (DHAR) can enhance the activity of the AsA-GSH cycle, thereby further improving the drought tolerance of wheat seedlings. LW-CNs are considered capable of scavenging multiple reactive oxygen species, thereby maintaining the equilibrium state of reactive oxygen species (Wang et al., 2025b). In this study, following the application of LW-CNs, the highly active APX efficiently oxidized AsA to MDHA. DHAR activity effectively assisted AsA in scavenging reactive oxygen species, whilst GSH could be reduced wwwwwvia glutathione reductase (GR), subsequently enabling DHAR to reduce DHA back to AsA. This process thereby sustained AsA’s capacity to scavenge reactive oxygen species. These results are consistent with the conclusions of relevant trials on maize and Brassica (Singh et al., 2020; Jiang et al., 2022). Overall, enhancing the antioxidant capacity of soybeans through nanoparticles represents a viable strategy for maintaining reactive oxygen species balance and improving soybean tolerance to high-temperature stress (**Figure 7**).

### 4.3 LW-CNs enhanced soybeans N absorption under heat stress

N is an essential nutrient element for plant growth and development, participating in the synthesis of organic compounds such as proteins, nucleic acids, chlorophyll, enzymes, and vitamins (Sharma et al., 2018; Du et al., 2020). After being absorbed by plants, N is reduced to ammonium nitrogen (NH_4_^+^), which is involved in the synthesis metabolism of amino acids. Previous studies have suggested that SiO_2_NPs enhance core metabolic pathways, particularly the tricarboxylic acid cycle, which plays a crucial role in maintaining cellular energy during adverse conditions in *Brassica napus* (Faisal et al., 2025). In maize, the combination of GO (graphene oxide) with seed coating agents enhances nutrient uptake by plants and promotes carbon and nitrogen metabolism in leaves (Zhang et al., 2025). In our research, soybean leaves treated with LW-CNs exhibited a higher NH_4_^+^ content under heat stress. This finding further corroborates that LW-CNs enhances the nitrogen utilization efficiency of soybeans during heat stress. The GS - GOGAT pathway plays a crucial role in regulating nitrogen assimilation (Miflin and Habash, 2002; Swarbreck et al., 2010; Fortunato et al., 2023). Our results demonstrated that LW-CNs treatment significantly elevates the activities of GS and GOGAT in soybean leaves, thereby strengthening the amino acid biosynthesis pathway. Additionally, the increased activity of GDH enhances the stress tolerance of soybeans. GPT can catalyze the reaction between Glu and pyruvate to produce alanine and α - ketoglutarate, facilitating the maintenance of energy metabolism under abiotic stress conditions (Forde and Lea, 2007; Labboun et al., 2009). GOT, on the other hand, can catalyze the reaction between Glu and oxaloacetic acid to yield aspartate and α - ketoglutarate, thus establishing a connection between nitrogen metabolism and the tricarboxylic acid cycle and coordinating the distribution of energy and nitrogen (Daubresse et al., 2006; Hildebrandt, 2018). In our study, the elevation of GPT and GOT activities mediated by LW-CNs foliar spraying contributes to the regeneration of tricarboxylic acid cycle intermediates (α - ketoglutarate), regulates energy homeostasis, and influences amino acid synthesis. Therefore, this process ensures that soybeans maintain normal metabolism under high-temperature stress, thereby mitigating damage to the soybeans.

### 4.4 The safety of nanomaterials is of great significance for increasing grain production

Nanoscience is an emerging and powerful field. It has found widespread application in agriculture, such as in fertilizers, pesticides, the mitigation of plant stress, and the enhancement of seed germination rates (Nair et al., 2010; Jiang et al., 2022; Kwaslema, 2024; Saeed et al., 2025). Among the numerous nanomaterials used in agriculture, industrially synthesized materials remain the mainstream. However, such materials not only significantly increase agricultural production costs but may also pose potential ecological and biosafety risks. Against this backdrop, the use of natural herbal plants to synthesize nanomaterials is gradually demonstrating its unique advantages and feasibility. Taking our research as an example, we use common Chinese herbal medicines such as licorice and goji berries as precursor materials, with a unit cost of only approximately 0.5 RMB per gram (Wang et al., 2025b), which is significantly lower than that of traditional industrial raw materials. This not only effectively reduces the raw material costs of agricultural nanomaterials but also provides new insights for the development of green agriculture. Therefore, nanomaterials extracted from herbal medicines are expected to play a significant role in enhancing crop production efficiency.

## 5 Conclusions

Based on our research findings, we describe the mechanism by which LW-CNs mitigate the effects of high-temperature stress on soybean plants. Research has revealed that LW-CNs treatment reduces malondialdehyde (MDA) content by enhancing antioxidant enzyme activity and scavenging excess reactive oxygen species (ROS). This process plays a pivotal role in soybean’s response to heat stress, particularly within the ascorbate-glutathione (AsA-GSH) cycle—a crucial pathway regulating the maintenance of reactive oxygen species homeostasis. A better understanding of the complex nitrogen metabolism can enhance the nitrogen utilization efficiency of soybeans, which is an important goal for achieving sustainable agriculture. Concurrently, LW-CNs enhance nitrogen assimilation in soybean by increasing the activity of Nitrogen assimilate enzymes such as GS, GOGAT, and GDH, GOT and GPT. Overall, the findings of our study provide more insights into the mechanisms behind herbal derived nanomaterials induced plant heat tolerance.

## Data Availability

The raw data supporting the conclusions of this article will be made available by the authors, without undue reservation.
